# Administration of Medicine to Children

**Published:** 1860-02

**Authors:** 


### ADMINISTRATION OF MEDICINE TO CHILDREN.

(From the Druggists Circular.)

M. Wahu having much to do with the diseases of children, wishes to impress upon practitioners the importance, in the case of important medicinal substances, of having them administered, whenever practicable, in their own presence. He also states some of the means he adopts to enable certain medicines to be more easily got down. Thus, the subnitrate of bismuth, in large doses, which is of such value in diarrhœa and the gastro-intestinal affections of children, often subsides to the bottom of the spoon when given in broth or milk; and it is much more readily taken also by children of about two years old when given in chocolate prepared in water, and thickened with tapioca or crumbs of bread. In this way seventy grains and more may be given night and morning. In the same way iron can be very readily given. Ratany and catechu, two precious drugs, the action of which, when in small doses, is soon manifested in children, can also very readily be given in this chocolate panada. Chocolate made with either milk or water, and flavored with canella or vanilla, is usually very readily taken by children, and its dark color facilitates the mixture of numerous colored medicinal substances, which would be observed by the little patients were they given in milk, broth, or any infusion. Ratany and catechu may also be well triturated and mixed with jelly, the flavor of which, while masking that of other substances, is very agreeable to children.

Sulphate of soda and sulphate of magnesia are substances very difficult to get even adults to take. The sulphate of soda may be often administered to children, by dissolving ten parts in 150 of unsalted beef-tea, and waiting until the child is sufficiently thirsty to swallow a cup of liquid almost without tasting it. For adults, the best is to dissolve the sulphate of soda or magnesia in exactly the quantity of hot water necessary for its complete solution. This is allowed to get cold, and a glass of pretty strong lemonade is prepared.—Holding a glass in each hand, that containg the salt is rapidly drunk, and then the lemonade is slowly drunk—masking the detestable taste of the purgative, and supplying each fluid to prevent its proving to irritating. Corsican moss is another substance which children take with difficulty: but if an infusion be made and strained, and then added to unsalted beef tea, it will be readily swallowed.

Calomel is one of the most difficult medicines to give, when children are too young to swallow pills, which is the case under six years of age. Incorporating it into honey is the best means—rinsing the mouth afterwards, to prevent any adhering to the gums. It should never be given in currant, or any other jelly; a death having occurred a few years since from the conversion of the calomel given in current jelly, into a bichloride. It is safest to prohibit any acid drink being taken on the day that calomel is given. Ipecac may be given either in the chocolate panada or in honey.

When it is impossible to give any medicinal substance by the mouth, it may be administered by the rectum, taking care first to empty the gut by tepid water or an emollient decoction, and that the bulk of the medicated enema does not exceed from four to six ounces, so that it may be retained and absorbed.

